# Design of Molecularly Imprinted Polymers Using Supercritical Carbon Dioxide Technology

**DOI:** 10.3390/molecules29050926

**Published:** 2024-02-20

**Authors:** Ana I. Furtado, Vasco D. B. Bonifácio, Raquel Viveiros, Teresa Casimiro

**Affiliations:** 1 LAQV–REQUIMTE, Chemistry Department, NOVA School of Science & Technology, NOVA University of Lisbon, 2829-516 Caparica, Portugal; ai.furtado@campus.fct.unl.pt (A.I.F.); raquel.viveiros@fct.unl.pt (R.V.); 2 iBB–Institute for Bioengineering and Biosciences and i4HB–Institute for Health and Bioeconomy, Instituto Superior Técnico, University of Lisbon, 1049-001 Lisboa, Portugal; vasco.bonifacio@tecnico.ulisboa.pt; 3 Bioengineering Department, Instituto Superior Técnico, University of Lisbon, Av. Rovisco Pais, 1049-001 Lisboa, Portugal

**Keywords:** affinity materials, rational design, computational chemistry, green chemistry, molecularly imprinted polymers, membranes, supported particles

## Abstract

The design and development of affinity polymeric materials through the use of green technology, such as supercritical carbon dioxide (scCO_2_), is a rapidly evolving field of research with vast applications across diverse areas, including analytical chemistry, pharmaceuticals, biomedicine, energy, food, and environmental remediation. These affinity polymeric materials are specifically engineered to interact with target molecules, demonstrating high affinity and selectivity. The unique properties of scCO_2_, which present both liquid– and gas–like properties and an accessible critical point, offer an environmentally–friendly and highly efficient technology for the synthesis and processing of polymers. The design and the synthesis of affinity polymeric materials in scCO_2_ involve several strategies. Commonly, the incorporation of functional groups or ligands into the polymer matrix allows for selective interactions with target compounds. The choice of monomer type, ligands, and synthesis conditions are key parameters of material performance in terms of both affinity and selectivity. In addition, molecular imprinting allied with co–polymerization and surface modification are commonly used in these strategies, enhancing the materials’ performance and versatility. This review aims to provide an overview of the key strategies and recent advancements in the design of affinity polymeric materials using scCO_2_.

## 1. Introduction

The two–time Nobel prize winner, L. C. Pauling, described that the secret of life is hidden in molecular recognition, which is the ability of one molecule to “recognize” another one through bonding interactions [1]. Since the beginning, researchers have been trying to mimic nature in the design of affinity synthetic materials. Polymers, dendrimers, chain polymers, and coordination polymers have appeared as very interesting materials that are suitable to replace natural molecules, such as aptamers and antibodies, which are very sensitive to handling, management, and storage [2,3]. However, these polymers lack the specific molecular recognition abilities that natural biomolecules have.

Affinity–driven synthetic materials, such as Molecularly Imprinted Polymers (MIPs), are tailor–made materials that can mimic the recognition of biological receptors [4]. MIPs are very appealing not only due to their molecular recognition ability but also due to the fact that they can surpass natural receptors in terms of robustness, chemical stability, durability, storage (no need for cold chain), binding affinity constants similar to natural molecules, and low production costs. 

MIPs are first produced via the formation of a complex between the template molecule of interest and the functional monomer(s) in the presence of a crosslinker agent and a porogen solvent to provide specific sites within the polymeric material that are physically and chemically complementary to the template. At the end of the process, the template is removed, leaving an empty cavity that can selectively bind the template molecule in the final MIP application. Different strategies can be used to prepare MIPs (non–covalent, covalent, semi–covalent, electrostatic/ionic, and metal center coordination), which only differ in the type and nature of the interactions between the template and functional monomer. The recognition ability is mediated by weak non–covalent hydrogen bonding interactions, ion–pairing, hydrophobic, or dipolar interactions. Moreover, traditional MIP polymerization techniques are in bulk, precipitation, emulsion, multi–step swelling, suspension, gelation, etc. Typically, in bulk polymerization, the obtained MIP needs to be crushed and sieved, a very time–consuming and laborious process, providing irregular particles in which the interaction sites are partially destroyed, compromising the molecular recognition process [5]. 

The global market of affinity materials, driven by polymers, is valued at USD 2.78 billion (2023), and it is expanding, being estimated to reach USD 4.14 billion (2028) [6]. In addition, there is an ever–increasing concern relating to the design of chemical processes allied to the minimization of environmental issues to reduce the generation of hazardous wastes. This is also aligned with the 2030 Agenda for Sustainable Development, which set out a 15–year plan to achieve the goals contributing to a safer planet [7]. The 12 principles of green chemistry and engineering can be followed to support cleaner and more sustainable choices in the whole life cycle of a product, from its design, production, and use to its final discarding [8]. 

Several conventional strategies have been developed involving the use of large quantities of solvents and intensive processing steps, contributing to the challenging process and application of MIPs at an industrial scale. On the other hand, several green and alternative solvents/technologies have emerged, strengthening the environmental sustainability of chemical processes and contributing to overcoming these drawbacks, such as supercritical fluids (in particular supercritical carbon dioxide–scCO_2_), ionic liquids, deep eutectic solvents, fluorous solvents, and mechanochemistry (solventless) [9,10,11,12,13]. 

ScCO_2_ is non–toxic, non–flammable, inert, odorless, can be easily removed without any additional energy input, and can be recycled, making it a scalable green alternative solvent/technology suitable for replacing conventional organic solvents typically used in polymer synthesis and processing. CO_2_ has an easily achieved and mild critical point (p_C_ = 73.8 bar; T_C_ = 31.1 °C). Above the critical point, CO_2_ combines the best properties of gas and a liquid, gas–like diffusivity and viscosity, and a liquid–like density, having a high mass transport capacity, high diffusivity, and zero surface tension. Additionally, the properties of CO_2_ can be simply adjusted by small changes in pressure and temperature [14]. Thanks to these properties, the potential of scCO_2_ has been continuously acknowledged in the synthesis and processing of polymers, such as dyeing, impregnation, and particle production, and in different polymerization techniques, including homogeneous polymerization, heterogeneous polymerization, precipitation polymerization, suspension polymerization, and emulsion polymerization [15]. Recent advancements in industrial applications in extraction, particle formation, micronization, encapsulation, impregnation, polymerization, and foaming underscore the viability and promise of scCO_2_–based chemistry [16].

**Figure 1:**
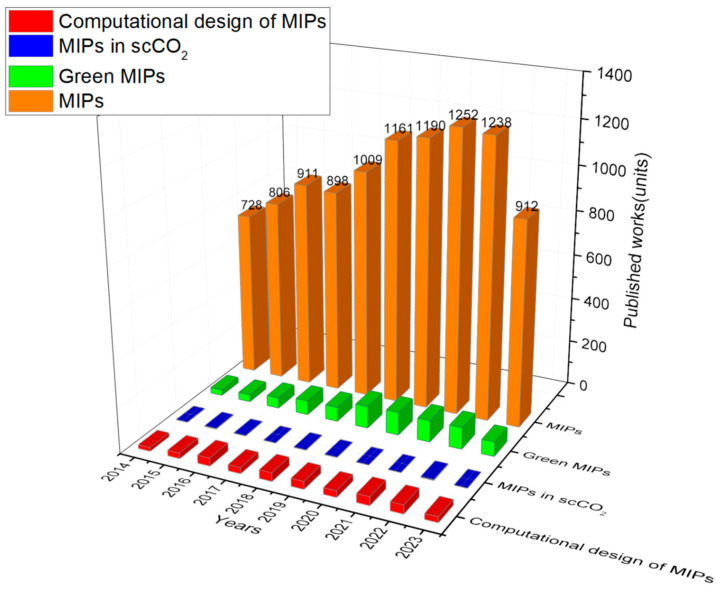
The figure shows the papers published in the field of Molecularly Imprinted Polymers (MIPs) divided into green MIPs, MIPs in supercritical CO_2_, and rational design MIPs using computational tools from 2014 to 2023. Source: Web of Science database derived from Clarivate [InCites. © Copyright Clarivate 2023. All rights reserved].

The development of MIPs using scCO_2_ also has many benefits compared to the traditional approaches since scCO_2_ is aprotic and has high mass transfer and diffusivity, which is not easily achievable when using organic solvents. MIPs are obtained via simple depressurization and cost–effective preparation [17] and have proven to be excellent greener alternatives as synthetic affinity materials for a wide range of applications [18]. Despite the fact that it is an excellent solvent to produce MIPs, the number of studiesexploring this technology is still limited, as can be seen in Figure 1. Therefore, herein, it is provided an overview of how scCO_2_ technology has been beneficial to the field of molecular imprinting, the design tools behind its production, and what is still needed to improve these processes for industrial scale to reach the market as sustainable, low–cost, tailor–made, and competitive materials.

## 2. Dry–Powder Molecularly Imprinted Polymers (MIPs)

To the best of our knowledge, the first MIP developed using scCO_2_ was reported by Duarte et al. in 2006 for drug delivery applications [19]. A molecularly imprinted poly(diethylene glycol dimethacrylate) (polyDEGDMA) was produced via free radical polymerization in scCO_2_ using carboxylic acid end–capped perfluoropolyether oil as a stabilizer for two different template molecules: salicylic acid and acetylsalicylic acid. An impregnation step was further performed, and the controlled release of the systems was evaluated. The release profiles of the systems studied showed a clear correlation between the amount of template imprinted and the impregnation amount, mainly for the system using acetylsalicylic acid as a template. This correlation was evident up to 3.8%w/w of the relative quantity of the drug in an impregnated sample. In the following years, other systems were reported such as propranolol, flufenamic acid, ibuprofen, bisphenol A, carbamazepine, and metronidazole. Table 1 summarizes the MIPs developed via free radical 24 h batch polymerization in scCO_2_ for several applications. As can be seen, most commercially available monomers are soluble in scCO_2_, namely those typically used in MIP synthesis. In all reported studies of scCO_2_–assisted MIP production, the removal of the template from the polymeric matrix after polymerization was also performed using scCO_2_ technology. Template desorption is a critical step in the molecular imprinting process to make the specific sites available for future re–binding in the final application. The template removal process with scCO_2_ was reported in the literature prior to the first synthesis of MIPs using scCO_2_. Ellwanger et al. reported the complete removal of the template from MIPs and proved that the use of scCO_2_ can increase the diffusion coefficient at least 10–fold compared to other methods [20]. For this step, the pre–synthesized MIP is introduced into a tubular column and subjected to the previous conditions. In some cases, when the template is highly soluble in some organic solvent, a small amount of cosolvent could be used by coupling a high–pressure cell with a cosolvent inside the tubular column so that the CO_2_ is bubbled through the cell containing the cosolvent, and the resulting solvent mixture traverses the MIP–packed column [17]. Typically, the most appropriate conditions for this step are 40 °C and 200 bar in a continuous flow mode for 3 h [12].

MIPs for separation applications just appeared in 2010, as reported by Soares da Silva et al. [21]. This study showed the development of a Boc–L–tryptophan–MIP for chiral separation. Micron–sized particles of poly(ethylene glycol dimethacrylate) (PEGDMA) and poly(*N*–isopropylacrylamide–*co*–ethylene glycol dimethacrylate) (P(NIPAAm–*co*–EGDMA))–based MIPs were successfully obtained at a high yield and packed into an HPLC blank column to evaluate their performance as a stationary phase in chromatography for the enantiomeric separation of L– and D–tryptophan. The co–MIP (NIPAAm–*co*–EGDMA based MIP) showed high potential for chiral separation (0.25–4 mM samples), obtaining a maximum capacity factor of 0.98 and a maximum retention enantioselectivity of 2.27. Further developments in MIPs for separation and wastewater treatment processes were mostly reported in the last decade, where better separation performance from MIP is verified with imprinting factors (*IF*) > 1.

In several cases, the MIPs synthesized using scCO_2_ have better performance in their applications compared to the conventional ones, with *IF*s typically higher than their counterparts’ systems in bulk polymerization [22,23,24,25]. *IF* is a common parameter in the MIP literature to evaluate the binding performance and imprinting effect of the materials. 

In a similar way to the design of conventional MIPs, the choice of functional monomers for a target template, the monomer ratio, the use of cosolvents, and the reaction conditions are critical factors that enhance the recognition performance of MIPs in their applications. In the literature on scCO_2_–assisted MIP synthesis, some optimization examples are described. For example, in the work of Viveiros et al., which describes acetamide (ACET)–MIP for Active Pharmaceutical Ingredient (API) purification processes [26], ACET–MIPs were produced using two different monomers, methacrylic acid (MAA) and methacrylamide (MAM), and the effect of the addition of a cosolvent, ACN (0.5 mL of ACN in 32.5 mL of scCO_2_), to polymer synthesis, was evaluated. The binding results revealed that both MIPs (MIP–MAA and MIP–MAM) have high affinity for ACET (*IF* > 1) in 10–250 ppm ACET organic solutions, but a significant effect on MIP performance was observed by the addition of a cosolvent in the polymerization step (maximum *IF* 4.5). In this work, a small amount of ACN was also added in the template desorption step (3 mL of ACN on the system pressurized with CO_2_ up to 210 bar), to ensure the complete removal of ACET. Another example is the work of Marcelo and Ferreira et al., who developed pH–responsive metronidazole–MIP using itaconic acid (ITA) as a functional monomer and EGDMA as a crosslinker for oral drug delivery [27]. In this work, the MIP performance was evaluated using two different crosslinking degrees, and, according to the drug release profiles, the MIP using the lowest crosslinking degree was able to load more drug (threefold more), and consequently, was the MIP that released more of the drug (twofold more) under physiological conditions. In several studies, the choice of the best functional monomer for the target template [12,23,26,28,29] and the use of more than one functional monomer [12,22,23,24] were also explored.

**Table 1:** MIP synthesis in scCO_2_.

Template	Nature of Template	Functional Monomer(s)	Crosslinker	Cosolvent	Initiator	T (°C)	p (bar)	*IF*	Application	Year	Ref.
Acetylsalicylic acid	Drug	-	EGDMA	-	AIBN AIBN	65	190	-	Drug delivery	2006	[19]
Salicylic acid	Drug			-							
(*R,S*)–Propranolol	Drug	MAA	DVB	-	AIBN	80	250	-	Separation	2006	[30]
				-		60	125	1.5			
				ACN	AIBN			15.7			
(*S*)–Propranolol				-				20.7			
Boc–L–tryptophan	Amino acid	-	EGDMA	-	AIBN	65	210	-	Separation	2010	[21]
		NIPAAm									
Flufenamic acid	Drug	MAA	EGDMA	-	AIBN	65	210	-	Drug delivery	2011	[28]
		NIPAAm									
Ibuprofen	Drug	DMAEMA	EGDMA	-	AIBN	65	210	-	Drug delivery	2011	[31]
Bisphenol A	Impurity	-	EGDMA	ACN	AIBN	65	210	2.3	Wastewater treatment	2012	[32]
Bisphenol A	Impurity	MMA; MAA	EGDMA	-	AIBN	70	300	5.8	Separation	2012	[25]
2,4–dichlorophenoxyacetic acid								5.3			
Acetaminophen	Drug	MMA; MAA	EGDMA	-	AIBN	65	300	-	Wastewater treatment	2013	[23]
		MMA; 4VP						3.8			
Aspirin	Drug	MMA; MAA						-			
		MMA; 4VP						3.9			
Carbamazepine	Drug	MAA	EGDMA	-	AIBN	65	300	3.8	Wastewater treatment	2014	[33]
Labdanolic acid	Natural drug	DMAEMA	EGDMA	ACN	AIBN	65	222	-	Separation	2014	[34]
Dibenzothiophene sulfone	Impurity	MAA	EGDMA	-	AIBN	65	222	1.3	Separation	2014	[35]
Gallic acid	Drug	MAA; MMA	EGDMA	THF	AIBN	65	300	3.6	Separation	2017	[22]
Acetamide	Impurity	MAA	EGDMA	-	AIBN	65	210	1.4	Separation	2017	[26]
		MAM		ACN				2.5			
		MAA		-				4.5			
		MAM		ACN				1.8			
Acetamide	Impurity	ITA	EGDMA	-	AIBN	65	210	1.3	Separation	2017	[29]
		HEMA						1.1			
Metronidazole	Drug	ITA	EGDMA	-	AIBN	65	210	-	Drug delivery	2018	[27]
Bisphenol A	Impurity	FMMA	EGDMA	-	AIBN	65	220	8.5	Wastewater treatment/ Sensing	2018	[36]
Benzamide	Impurity	MAM	EGDMA	-	AIBN	65	210	1.2	Separation	2018	[37]
Pivalamide								1.1			
Vanillic acid	Drug	MAA; MMA	EGDMA	THF	AIBN	65	300	2.7	Separation	2021	[24]
Cholesterol	Steroid	MAA	DVB	DMF	AIBN	65	280	-	Catalysis	2022	[38]
4–Dimethylaminopiridine	Impurity	MAA	EGDMA	-	AIBN	65	210	1.7	Separation	2023	[39]
L–leucine	Amino acid	2VP	EGDMA	EtOAc	V–65	45	200	12.0	Separation	2023	[12]
		AM						3.9			
		2V; AM						2.2			

The MIPs synthesized using scCO_2_ also proved to be suitable for the development of disposable and cost–effective MIP–based sensors. In Rebocho et al. [36], the development of a MIP–based disposable sensor for Bisphenol A (BPA) is described using ferrocenylmethyl methacrylate (FMMA) as a functional monomer and EGDMA as a crosslinker. The performance of the MIP as an electrochemical sensor was studied using commercial carbon screen–printed electrodes in the presence of BPA via differential pulse voltammetry, and the results show the successful detection of the BPA characteristic irreversible oxidation peak and the increase in the current intensity response with BPA concentration (4.7–8 nM).

In 2022, Viveiros et al. reported the first MIP developed using scCO_2_ for catalysis purposes [38]. In this work, a 2,2,6,6–tetramethyl–1–piperidinyloxy (TEMPO)–MIP catalyst was obtained after the template cleavage from the matrix, and the oxidation of the N–H groups enabled available TEMPO moieties within the MIP. The oxidation of benzyl alcohol, 5α–cholestan–3β–ol (cholesterol), and cholic acid was fast, in high yield, and with selective oxidation capacity, achieving close to 100% oxidation conversion after 8 min. 

MIPs synthesized using scCO_2_ for API separation processes as potential materials for demanding late–stage purification in pharmaceutical processes were also reported [39]. In this work, 4–dimethylaminopiridine (DMAP)–MIP was produced using MAA as a monomer, EGDMA as a crosslinker, and AIBN as a free radical initiator. DMAP is a genotoxic impurity from API crude mixtures. The DMAP extraction efficiency was evaluated via dynamic binding experiments using a 104 ppm DMAP crude solution, obtaining an *IF* of 1.7 and a recovery of 1004.6 µmol DMAP/g API. The most recent reported work on MIP synthesis in scCO_2_ was focused on biopurification processes. Furtado et al. reported the development of amino acid–MIPs [12]. In this work, a rational design using QM/MM calculations was followed to select the most appropriate monomers for the amino acid L–leucine (LEU) as a template. According to the experimental results obtained in scCO_2_–assisted polymerization systems, the LEU–MIP with the highest molecular recognition ability for the target molecule was obtained using 2–vinylpyridine (2VP) as a functional monomer, EGDMA as a crosslinker, and V–65 as a free radical initiator. A significant *IF* of 12 and a binding capacity (*Q*) of 27 mg LEU/g MIP was obtained in a 0.5 mg LEU/mL aqueous solution. In both works, the need for cleaner processes was highlighted, as well as more specific and cost–effective material solutions, such as these MIPs, since affinity, efficiency, and scale–up possibilities are critical assets in current purification processes. Furthermore, the integration of computational methods holds the potential to unlock strategies for designing MIPs (see Section 4) and accelerate the process of obtaining cost–effective, tailor–made MIPs for a wide range of templates.

## 3. Molecularly Imprinted 3D Porous Structures and Supported Devices

Research on imprinted 3D porous structures and supported particles has been growing since 2007, opening up the design of new affinity–driven polymeric formats (see Table 2). Molecularly imprinted membranes (MIMs) stand out prominently among 3D porous structures. All MIM works using scCO_2_ were developed through the phase inversion method, employing scCO_2_ as a non–solvent, with the casting solution pre-mixing in an organic solvent.

PSMA–molecularly imprinted membranes were prepared for uracil in several solvents (DMF, DMSO, and NMP) using two different temperatures (35 and 50 °C) [40]. The membranes prepared at 50 °C had higher affinity than the membranes prepared at 35 °C (12.6 vs. 9.2 μmol g^−1^, respectively), and a better binding performance was obtained when using DMSO in the casting solution. In this study, it was verified that the increase in temperature increased uracil solubility in PSMA, resulting in the formation of a higher number of homogenous imprinted microcavities. Related to the organic solvent cast, differences in the MIM pore morphology were verified when different organic solvents were used, where large pore size and slightly isolated pores were obtained when using NMP, contrary to interconnected pores obtained with DMF, and the greater number of interconnected pores obtained with DMSO, increasing its permeability and resulting in better performance in the binding assays. In further work, PA6/PSMA–composite MIMs were developed toward oleanolic acid under different temperature and pressure conditions (35 to 50 °C and 12 to 17 MPa) [41]. The MIMs produced using a mass ratio of PSMA and oleanolic acid of 6:1 at scCO_2_ conditions of 40 °C and 150 bar had the best performance, with an oleanolic acid adsorb rate of 50% and a purity of 96%. According to this study, as the temperature rises, the CO_2_ density decreases, consequently decreasing the solubility of the system in scCO_2_, but, with the increase in temperature, the vapor pressure of the system increases, contributing to the increasing solubility. The effect of temperature on phase inversion using scCO_2_ processes is quite complex and strictly dependent on the system used. In addition, at lower temperatures, the DMF used in the casting solution could not be well dissolved in CO_2_ fluid, negatively affecting the imprinting process in terms of the number and homogeneity of the imprinted microcavities. Therefore, the optimum temperature for this system (OA; PSMA; DMF) was identified as 40 °C. The same behavior was found with the variation in the pressure of the system, where at higher and lower pressures, the performance of the resultant MIM is conditionate, with 150 bar being identified as the optimum pressure. At lower pressures, a decrease in DMF dissolution is expected in the casting solution, negatively affecting the imprinting process, but at high pressures, it could negatively affect the membrane structure, destroying pores or disturbing the interaction between PSMA and the template. Finally, the mass ratio between the polymer and the template is also an important factor in terms of the morphology and properties of the MIM. It was verified that with the increase in PSMA, the surface thickness of the resultant MIM increased, but the porosimetry decreased, consequently decreasing membrane permeability, which is an important property in terms of its applications.

**Table 2:** Imprinted 3D porous structures and supported devices.

MIP–Based Membranes	Template	T (°C)	p (bar)	Reaction Time (h)	Cosolvent	*IF*	Application	Year	Ref.
PSMA	Uracil	35 to 50	160	2	DMF or DMSO, or NMP	5.0	Separation	2008	[40]
PA6/PSMA	Oleanolic acid	35 to 50	120 to 170	1.5 to 2.5	THF	1.1	Separation	2011	[41]
Poly(MAA–*co*–EGDMA)	Bisphenol A	45	200	3	DMF	1.3	Separation	2012	[42]
Poly(DM–*co*–EGDMA)	Bisphenol A	45	200	3	DMF	2.1	Separation	2012	[32]
**MIP–supported particles**									
CdTe	Bisphenol A	65	280	24	-	1.3	Sensing	2014	[43]
Large MIP–layered silica core–shell beads	Acetamide	65	210	24	-	-	Separation	2017	[44]

Moreover, scCO_2_–assisted phase inversion membranes from poly(MAA–*co*–EGDMA) and poly(DM–*co*–EGDMA)–MIPs were developed for BPA removal via non–covalent and semi–covalent imprinting methodologies, respectively [32,42]. In this semi–covalent approach, a template–containing monomer, Bisphenol A dimethacrylate, was used. In the end, the Bisphenol A molecule was cleaved from the polymeric matrix via hydrolysis with tetrabutylammonium hydroxide (*n*–Bu_4_OH), also in a supercritical environment, taking advantage of the high diffusivity of scCO_2_.

Most systems that use this approach use polymerizable acids containing unstable ester groups as functional monomers, and the template containing alcohol groups is used to form the covalent bonds since their cleavage is facilitated through a hydrolysis step [45]. Both materials, Poly(MAA–*co*–EGDMA) and Poly(DM–*co*–EGDMA), toward bisphenol A, were synthesized at 45 °C and 200 bar with a CO_2_ flow of 9.8 g min^−1^ for 3 h using a casting solution with a 30 wt% polymer blend consisting of 70:30 of PMMA and MIP particles in 5 mL of DMF. In the end, the system was slowly depressurized over 20 min, and a thin homogeneous membrane was obtained. The non–covalent imprinted and semi–covalent MIM could adsorb 1.3 and 2.1 times more BPA than the analogous control materials. In this case, the semi–covalent MIM approach is highlighted. According to the different designs behind the previous MIM systems, it can be concluded that factors such as temperature, pressure, the concentration of the polymer, and the casting solvent are important factors in achieving effective imprinted microcavities, as well as membrane morphology with properties that ally with its further applications (e.g., porosimetry, permeability, and robustness). These previous factors are also included in the design of other types of polymeric membranes for affinity separation processes [46].

Other devices utilizing scCO_2_ as a solvent have also been explored. MIP–supported particles have been demonstrated to be a very appealing strategy in terms of modifying and improving the core materials for enhanced performance. A quantum dot (QD) molecularly imprinted sensor to BPA was developed using scCO_2_ (CdTe@MIPs), in which CdTe QDs were previously functionalized in a conventional way and further coated with poly(MAA–*co*–EGDMA) with an affinity toward BPA under scCO_2_. These MIPs take advantage of the exceptional QDs optoelectronic properties. Highly sensitive and selective fluorescence quenching was achieved for a well-defined and extremely low range of concentrations (4–10 nM) [43]. Likewise, a gravity–driven purification device for pharma purification has been developed. Large core beads were pre–functionalized using two different green strategies (scCO_2_ and plasma technology). The surfaces of the silica beads were functionalized using the *grafting from* strategy using argon plasma, and then a poly(MAA–*co*–EGDMA) layer was formed with an affinity toward ACET using scCO_2_ technology, producing large core–shell beads [44]. The imprinted particles were able to remove 100% of ACET with a minimal loss of API, which is a very interesting result from an industrial point of view.

## 4. Material Design Tools

Over the past decade, rapid growth in advanced materials research has been seen, attributing a new importance to the role of the computational design of materials for deep understanding and prediction of the behavior of these materials [47]. This has been mainly driven by the increase in computational power and by the search for new and more efficient computational approaches, which has enabled the analysis and processing of increasingly complex information, such as larger systems, extreme conditions, and hard simulations involving long periods of time. In addition, the use of computational methods in pre–design and further experimental material synthesis significantly reduces the cost and the time involved in the process. The computational design of affinity materials, such as MIPs, is mainly focused on their molecular interactions to understand their unique structure–derived properties using approaches such as ab initio methods, force–field techniques, and machine learning (ML), establishing structure–property relationships in the design of innovative materials and enhancing their performance. Most of the computational approaches use the following: (1) quantum mechanics (QMs) or molecular mechanics (MMs) to quantify the interaction between the receptor and the ligand; (2) structure–based virtual screening to evaluate different ligands and select the most promising ones; and (3) molecular dynamics to simulate multicomponent systems, taking time and the dynamic effects into consideration [48,49,50]. In recent years, the design of experiments (DOE) has been left aside due to time–consuming laboratory work; however, the use of DOE incorporated into big data analysis allied to ML has been described as the next scientific paradigm in materials design [51]. Each method has advantages and disadvantages, and different assumptions are made in each method; therefore, a critical understanding of the results is always required. For this reason, experimental validation is an essential part of the process to confirm the performance of the proposed computational method.

Alternatively, strategies that use more than one method are being increasingly embraced, such as combinations of QM–MM, QM–MD, MM–MD, and QM–MM–MD, to better describe multi–molecular systems. The strategy adopted is usually according to template size and properties and libraries screened, among other features of the system. Even more, molecular/atomistic simulations combined with ML approaches have gained popularity in recent years to achieve faster, high–accuracy simulations and enable predictions of properties that cannot be simulated, such as biological, larger size and larger simulation time systems [52].

Despite the computational growth in the field of materials design and its inherent advantages, the application of these tools in systems involving supercritical fluids is still challenging. One of the reasons is the variability in terms of strategies, conditions, systems used in MIP development, and the lack of experimental data concerning the use of scCO_2_ for validation. The use of computational tools in scCO_2_ systems mainly focuses on the prediction of compound properties under scCO_2_, such as diffusivity, solubility, and density, which are highly important data in terms of extraction, impregnation, and other processing processes using this technology [53]. It is essential to have knowledge of that information for MIP synthesis since a single homogeneous phase prior to polymerization under supercritical CO_2_ is a critical parameter in obtaining homogeneous MIPs with effective recognition capability. These properties prediction studies could be behind the progress in computational MIP protocols allied to scCO_2_ technology. Many of these studies predominantly rely on MD simulations to gain insights into understanding solute–solvent intermolecular interactions. This deep understanding is achieved by representing the solvent as a continuum, employing empirical models, and using equations of state to calculate diffusion coefficients and solubilities of compounds in scCO_2_ [53]. The book by Gupta and Shim, as well as other works in the literature, provide summaries of solubility data in relation to scCO_2_ [53,54,55,56,57]. In general, small and non–polar molecules exhibit higher solubility in scCO_2_. Notably, the field of ML has also witnessed significant expansion in the last two years (more than 30 reported studies) [58,59].

Computational studies for MIP systems considering scCO_2_ as a solvent are quite limited, and just one is reported in the MIP literature, conducted by Viveiros et al. [29]. Acetamide (ACET)–MIP systems were studied using the *SYBYL*^TM^ software for MD, MM as a refining step, and a virtual library of 25 commonly screened monomers against the template (acetamide) using the LEAPFROG™ algorithm. The study was conducted to understand the effect of CO_2_ on template–monomer (T:M) interactions. The reported results showed that the T:M complex using itaconic acid (ITA) (acid monomer) or 2–hydroxyethyl methacrylate (HEMA) (neutral monomer) as a monomer (M) was not negatively affected by the presence of CO_2_. On the other hand, the T:M complex with bisacrylamide (BIS) (basic monomer) was destabilized when CO_2_ was added. This destabilization emerges from CO_2_ interactions with specific atoms in BIS that should form favorable interactions with the template. This work also proposed a set of monomers, namely methacrylamide (MAM), vinylimidazole, 2–vinylpyridine (2VP), epichlorohydrin, 4–vinylpyridine (4VP), and methyl methacrylate (MMA), which were selected based on their binding energy with CO_2_. This selected list indicated the potential influence of CO_2_ on these monomers, possibly impeding their binding to the template and, consequently, destabilizing the T:M complex. In the end, computational studies revealed a positive trend between the virtual and experimental results. In another attempt at more accurate computational correlation studies using scCO_2_ as a solvent, Furtado et al. developed QM calculations using *Gaussian* 09 software and MM using *Autodock Vina* to select the most appropriate monomers [12]. According to the binding energy calculations of the T:M complexes, no trend between the theoretical and practical results were verified, indicating that QM calculations without taking the effect of solvent into account is not enough to describe and understand the MIP systems in scCO_2_. However, in this reported work, it is mentioned that T:M complexes with electric dipole moment (EDM) lower and closer to the EDM of scCO_2_ could reflect a more stable conformation of its T:M complex, consequently envisaging better molecular recognition performance. In conclusion, both studies underscore the crucial importance of achieving compatibility among the monomers, templates, and solvents for an effective imprinting process and forming MIP. Even so, as was previously noted in both reported works, just looking for the CO_2_ effect can be misleading, and a more holistic approach needs to be followed since other molecules (e.g., crosslinker, cosolvent, etc.) and parameters (e.g., pressure and temperature) are involved in the MIP system.

Further investigation and validation of the computational methods are necessary to better understand and obtain accurate MIP systems using scCO_2_. Additional research in this field is essential to bridge the existing data gap, encompassing both experimental and theoretical aspects. For example, monomer libraries could be extended in terms of compatibility in the solvent and target template, including aspects such as the solubility of monomers in the chosen solvent (in this case, scCO_2_ solubility data), the potential for undesirable chemical reactions (undesirable interaction with CO_2_ or other molecules that are not directly involved in the imprinting process, such as initiators and crosslinkers), and the impact of the solvent on the overall polymerization process [48]. Moreover, this type of standardized data could inspire ML that combines various computational protocols with experimental data, enabling quicker and more accessible predictions for all monomers and templates within specific solvents, as well as information about other features that impact polymer performance, including solvent properties (such as temperature, pressure, and pH) and monomer ratios (e.g., saturation conditions).

## 5. Conclusions

This overview highlights the significant advancements and promising prospects of MIP synthesis using scCO_2_ technology. scCO_2_ technology not only offers a green and sustainable alternative to conventional polymerization processes but also enhances the efficiency and performance of MIPs. There are numerous advantages to using scCO_2_, such as its non–toxicity, high diffusivity, and recyclability, resulting in ready–to–use and solvent–free materials with scale–up potential and contributing to the sustainable production of MIPs with molecular recognition sites. The synthesis of MIPs in scCO_2_ and their 3D porous structures and supported devices has already demonstrated their high potential across various applications, ranging from drug delivery, catalysis, separation processes, and sensor development. The key challenges behind scCO_2_–assisted MIP production is related to the limited solubility of some molecules (e.g., polar molecules and more biological molecules) in scCO_2_. As can be seen in this review, several potential solutions could be found by using strategies that include the addition of a small amount of organic cosolvent or opting for a more compatible monomer for the template and scCO_2_. This review also emphasizes the need for a holistic approach that considers various factors beyond the influence of CO_2_ as a solvent. Additionally, the integration of computational methods, including QM, MD, and ML, plays a crucial role in the optimization of MIP design. Overall, the presented works showed the potential of scCO_2_–assisted MIP synthesis as a sustainable, cost–effective, and tailor–made solution for diverse applications in the growing field of affinity–driven synthetic materials.

## Data Availability

Not applicable.

## Acronyms

ACET—Acetamide
ACN—Acetonitrile
AIBN—Azobisisobutyronitrile
AM—Acrylamide
API—Active Pharmaceutical Ingredient
BIS—Bisacrylamide
BPA—Bisphenol A
CdTe—Cadmium telluride
DM—Dimethacrylate
DMAEMA—2–(Dimethylamino)ethyl methacrylate
DMAP—4–Dimethylaminopiridine
DMF—Dimethylformamide
DMSO—Dimethyl sulfoxide
DOE—Design of experiments
DVB—Divinylbenzene
EDM—Electric dipole moment
EGDMA—Ethylene glycol dimethacrylate
EtOAc—Ethyl acetate
FMMA—Ferrocenylmethyl methacrylate
HEMA—2–Hydroxyethyl methacrylate
*IF*—Imprinting factor = binding capacity from MIP divided by the binding capacity from NIP
ITA—Itaconic acid
LEU—L–Leucine
MAA—Methacrylic acid
MAM—Methacrylamide
MD—Molecular dynamics
MIM—Molecular imprinted membrane
MIP—Molecular imprinted polymer
ML—Machine learning
MM—Molecular mechanics
MMA—Methyl Methacrylate
NIP—Non–Imprinted Polymer
NIPAAm—*N*–Isopropylacrylamide
NMP—*N*–Methyl–2–pyrrolidone
p—Pressure
PA6—Polyamide–6
pc—Critical pressure
polyDEGDMA—Poly(diethylene glycol dimethacrylate)
PSMA—Poly(styrene-*co*-maleic acid)
*Q*—Binding capacity
QDs—Quantum dots
QM—Quantum mechanics
scCO_2_—Supercritical carbon dioxide
T—Temperature
Tc—Critical temperature
T:M—Template−monomer
TEMPO—2,2,6,6–Tetramethyl–1–piperidinyloxy
THF—Tetrahydrofuran
V–65—2,2’–Azobis(2,4–dimethylvaleronitrile)
2VP—2–Vinylpyridine
4VP—4–Vinylpyridine
